# Central Retinal Artery Occlusion in a 6-Year-Old Child with an Acute Chickenpox Infection: A Case Report

**DOI:** 10.3390/jcm14248685

**Published:** 2025-12-08

**Authors:** Dunja Bajtl, Tvrtka Benašić, Jelena Petrinović-Dorešić, Nenad Vukojević, Dubravka Biuk, Ivona Barać, Sanja Perić

**Affiliations:** 1 University Eye Department, University Hospital Centre Osijek, 31000 Osijek, Croatia; dunja.bajtl@kbco.hr (D.B.);; 2 Faculty of Medicine, Josip Juraj Strossmayer University of Osijek, 31000 Osijek, Croatia; 3 University Eye Department, Reference Centre of the Ministry of Health of the Republic of Croatia for Paediatric Ophthalmology and Strabismus, Reference Centre of the Ministry of Health of the Republic of Croatia for Inherited Retinal Dystrophies, University Hospital “Sveti Duh”, 10000 Zagreb, Croatia; 4 University Eye Department, University Hospital Centre Zagreb, 10000 Zagreb, Croatiapericsanj@gmail.com (S.P.); 5 School of Medicine, University of Zagreb, 10000 Zagreb, Croatia; 6 University Department of Surgery, University Hospital Centre Osijek, 31000 Osijek, Croatia

**Keywords:** antibody, anticardiolipin, central retinal artery occlusion, chickenpox, child

## Abstract

**Background:** Central retinal artery occlusion (CRAO) is an ophthalmic emergency attributed to a vessel occlusion with an embolus or a thrombus and may occur during the hypercoagulable state, inflammation, or vasculitis. CRAO may occur in children; however its incidence is very rare. Most pediatric cases have detectable etiologies. **Case Presentation:** We describe the case of an otherwise-healthy six-year-old female, who presented with the sudden and complete vision loss of the left eye lasting over twelve hours after a six-day chickenpox exanthema, followed by a high fever. All the ophthalmological, laboratory, and instrumental investigations led to the diagnosis of a left CRAO. Laboratory testing was unremarkable except for the transient elevation of D dimers (1363 µg/L), IgM anticardiolipin antibodies (238.5 CU), and IgG anti-beta-2 glycoprotein-1 antibodies (76.1 CU) on admission. Thrombolytic treatment was not exerted because of late presentation to the hospital. Treatment with steroids, antiviral medications, antibiotics, and anticoagulants was obtained, but the visual outcome was poor during the hospitalization and at the last follow-up. We could not ascribe features of this case to any etiological condition apart from the documented ongoing chickenpox infection. **Conclusions:** This is the first case report of CRAO in a child with transient aPL elevation and acute chickenpox infection.

## 1. Introduction

Central retinal artery occlusion (CRAO) is an ophthalmic emergency attributed to a vessel occlusion by an embolus or thrombus formation because of a hypercoagulable state, emboli from cardiac valvular disease, vasculitis, or inflammation. Most pediatric cases have detectable etiologies: cardiac valvular disease or vasculitis [1,2,3,4]. In several case reports, CRAO in children occurs in combination with central retinal vein occlusion, for example, in patients with nephrotic syndrome or non-Hodgkin lymphoma [3,4].

The hypercoagulable state can be primary (inherited disorders of factor V Leiden, prothrombin gene mutation, protein C and S deficiency, and antithrombin III deficiency) or secondary (cancer, smoking, obesity, pregnancy, major trauma or surgery, inflammatory or autoimmune disorders, prolonged immobilization, and certain medications) [5,6].

Viral infections can cause a transient hypercoagulable state because of mechanisms like direct cell damage, inflammation, and the body’s immune response [5]. Some viral infections can trigger the transient but detectable elevation of antiphospholipid antibodies (aPL) [6,7].

Antiphospholipid syndrome (aPS) is an autoimmune disorder caused by auto-antibodies being directed to phospholipids, which are components of the cell membrane. Individuals with antiphospholipid syndrome have a high risk of developing arterial and venous thrombosis, as well as obstetric complications like a miscarriage [8].

The 2023 ACR/EULAR aPS classification criteria include an entry criterion of at least one positive aPL test within 3 years of identification of an aPL-associated clinical criterion, followed by six additive clinical criteria (macrovascular venous thromboembolism, macrovascular arterial thrombosis, microvascular, obstetric, cardiac valve, and hematologic) and two laboratory criteria (lupus anticoagulant [LAC] functional coagulation assays, and solid-phase enzyme-linked immunosorbent assays for IgG/IgM anticardiolipin [aCL] and/or IgG/IgM anti–β2-glycoprotein 1 antibodies [aβ2GP1]). At least two positive aPL tests performed at least 12 weeks apart are required for the diagnosis of aPS [9].

To the best of our knowledge, this is the first report presenting unilateral CRAO with elevated aPL and acute chickenpox infection.

The aim of this article is to present the case of a child suffering from unilateral CRAO with an acute chickenpox infection and transient antiphospholipid antibodies (aPL) elevation.

## 2. Detailed Case Report

A 6-year-old female was admitted to University Hospital Center Osijek complaining of diminished vision in her left eye (LE). The symptoms emerged over 12 h before the admission with moderate pain, followed by monochromatic vision and sudden vision loss in the LE. The patient’s sister was COVID-19 positive 2 months before the onset of ocular symptoms in our patient. The patient did not display any COVID-19 related symptoms and was negative (COVID-19 fast antigen) at the time of the sibling’s active infection and via PCR test upon admission to the hospital. There was no history of COVID-19 vaccination.

**Figure 1:**
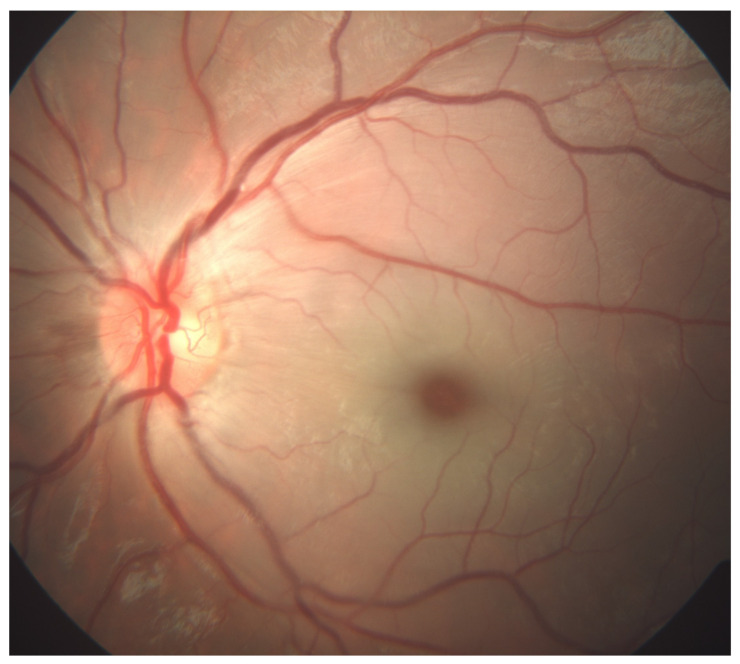
Color fundus photography (FA, Zeiss FF 450 plus, VISUPAC software, Jena, Germany) of a six-year-old patient’s left eye with prominent features of central retinal artery occlusion: retinal whitening, a cherry-red spot, and arteriolar attenuation without distinguishable emboli.

Six days prior to the ocular symptoms, chickenpox exanthema emerged. The patient was highly febrile for the first few days of the infection. There was no history of recent tick exposure. Visual acuity upon examination measured using the Snellen chart was 6/6 on the right eye (RE) and light perception on the LE. Pupil examination revealed a 1+ relative afferent pupillary defect of the LE. The anterior segment, intraocular pressure, ocular motility, orthoptic examination and ocular ultrasound were unremarkable. Dilated fundus examination displayed a normal fundus of the RE. The LE exhibited an appearance of CRAO: retinal whitening, a cherry-red spot, and arteriolar attenuation without distinguishable emboli (Figure 1).

**Figure 2:**
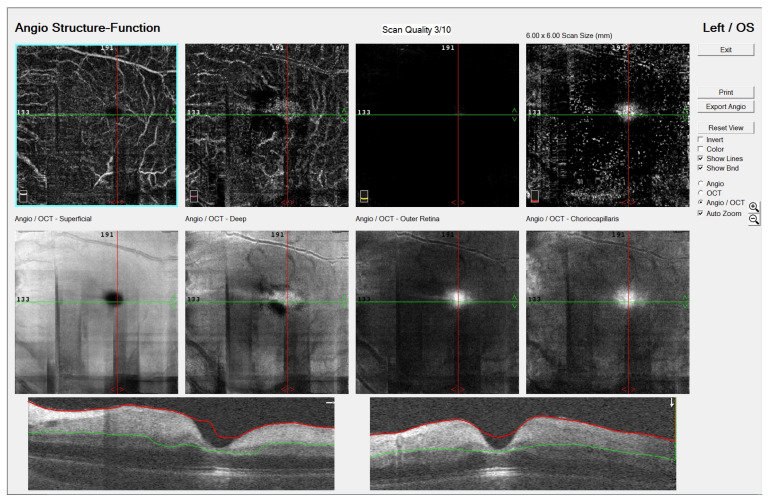
Optical coherence tomography angiography (OCTA, OptoVue Avanti XR version 2016.1.0.26, Optovue Inc., Fremont, California, United States of America) image of a six-year-old patient’s left eye with central retinal artery occlusion: disruption of the superficial and deep capillary plexus with decreased vascular perfusion. Optical coherence tomography (OCT) displayed hyper-reflectivity of inner and hypo-reflectivity of outer retinal layers with increased central macular thickness and loss of organized layer structure.

Optical coherence tomography angiography (OCTA, OptoVue Avanti XR version 2016.1.0.26, Optovue Inc., Fremont, California, United States of America) revealed normal retinal circulation in the RE, while the LE showed disruption of the superficial and deep capillary plexus with decreased vascular perfusion. Optical coherence tomography (OCT) displayed the hyper-reflectivity of the inner and the hypo-reflectivity of the outer retinal layers with increased central macular thickness and a loss of organized layer structure (Figure 2).

On the same day that the ocular symptoms emerged, magnetic resonance (MR) imaging, including MR angiography, was performed. Unfortunately, the visualization of the ophthalmic artery was unsuccessful because of technical difficulties. Laboratory testing results at admission corroborating with viral infection are presented in Table 1. The X-ray images of the heart and lungs were normal.

Additional laboratory testing results from the hospital stay are presented in Table 2. Laboratory testing outside of normal values included elevated IgM aCL and IgG aβ2GP1: 238.5 CU and 76.1 CU, respectively. LAC was not measured because of technical difficulties. Cerebrospinal fluid (CSF) showed some pleocytosis (leucocytes 19 cells/mm^3^), but the cultures test was negative for all infectious agents including herpes simplex virus 1 and 2, Cytomegalovirus, and Varicella Zoster virus.

**Figure 3:**
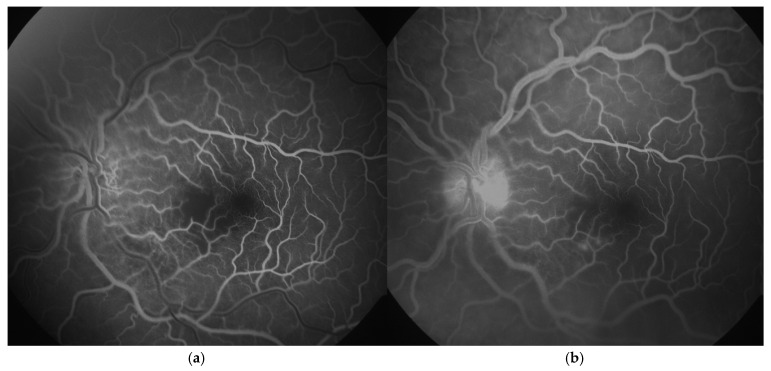
Fluorescein angiography (FA, Zeiss FF 450 plus, VISUPAC software, Jena, Germany) images of a six-year-old patient’s left eye with central retinal artery occlusion: (**a**). arteriovenous phase (reduced capillary perfusion in the papillomacular bundle and the inferonasal part of the macula); (**b**). late phase (delayed filling with slight dye leakage from retinal capillaries in the affected areas).

Both a pediatric immunologist and an infectologist were consulted because of the strong suspicion of aPS and chickenpox. Therapy included intravenous acyclovir (7 days) combined with methylprednisolone 40 mg/kg for three days followed with 2 mg/kg oral prednisone for one month, ceftriaxone (12 days), vitamin D, and gastroprotection. No evidence of connective tissue disease was found. Considering that the symptoms started over six hours earlier than the admission, thrombolytic treatment was not exerted. Anticoagulant therapy (enoxaparin 10 mg/dan) was therefore initiated. Fluorescein angiography (FA, Zeiss FF 450 plus, VISUPAC software, Jena, Germany) was performed four days after the admission to the hospital and revealed reduced capillary perfusion in the papillomacular bundle and the inferonasal part of the macula, with delayed filling and leakage from retinal capillaries in the affected areas (Figure 3a,b). Echocardiography revealed a normal heart structure, without valve disease.

The child did not display any other symptoms except vision loss and chickenpox infection. The patient recovered from the chickenpox infection without non-ocular complications.

Throughout the ophthalmological follow-up, LE blindness persisted with no improvement, as well as the extreme reduction in the retinal nerve fiber layer (RNFL) at the OCT examinations at the 3-month and 5-month follow-ups. IgM aCL displayed conversion to a weak positive value one month after the admission (29.7 CU).

## 3. Discussion

CRAO is a condition typically affecting the older population, with an estimated incidence lower than 1:50,000 in people under 30 years old. Rare cases in children have been reported [1,2,3,4,10,11,12].

In our case, antiphospholipid antibodies (aPL) were detected as transiently elevated. aPL could be a potential biomarker for CRAO in children, especially for patients with recent viral infections.

### 3.1. Antiphospholipid Antibodies and CRAO

The most frequently described thrombophilia-related risk factor for CRAO is the occurrence of aPL, including LAC, aCL, and/or aβ2GPI antibodies, which was observed in more than one third of adult CRAO patients in a study by Dziedzic et al. [13]. On the contrary, antithrombin deficiency was not associated with CRAO in this study [13]. Similar studies have conflicting results [14,15,16]. Glueck et al. [14] reported higher LAC parameters in CRAO patients; however, aCL was not elevated. We did not find reported cases of triple aPL positivity in CRAO patients nor reports on aPL positivity in children with CRAO.

### 3.2. Relationship of Elevated Antiphospholipid Antibodies and Viral Infections

Several infections have shown the ability to trigger transient aPL elevation [6,7]. The most common preceding infection is viral with positive aCL antibodies. Parvovirus B19, cytomegalovirus, VZV, human immunodeficiency virus, hepatitis, and several bacteria are the most frequently associated with aPL elevation [15,16]. Vaarala et al. collected paired serum samples from 149 young adult patients with acute infections and showed a significant transient elevation of aCL levels in 20% of patients with chickenpox [17]. The exact mechanisms underlying the development of aPL following VZV infections are not completely understood. However, it is hypothesized that molecular mimicry or nonspecific immune activation triggered by the viral infection may lead to the production of aPL. Endothelial injury caused by VZV infection may contribute to a pro-thrombotic state in susceptible patients [18]. Gall et al. reported a case of a 40-year-old man with acute chickenpox infection, elevated aPL (aCL and aβ2GP1), dural venous sinus thrombosis, and pulmonary embolus (PE) [19]. Several other cases were reported connecting transient aCL elevation in varicella cases to thrombotic events, predominantly in veins [20,21,22,23].

### 3.3. Stroke in Children: Viral Infection as a Suspect

Stroke is the most similar entity to CRAO. Besides trauma, viral infections have been identified as a trigger for stroke in children [23]. Two subtypes of childhood stroke are ischemic: arterial ischemic stroke (AIS) and cerebral sinovenous thrombosis [24]. A large proportion of children with AIS suffer from another disease that predisposes them to stroke, for example, congenital heart disease, sickle cell disease, or a range of genetic disorders [24]. Cerebral arteriopathy is an important cause of childhood AIS, accounting for approximately half of all cases. Focal cerebral arteriopathy of childhood (FCA) is described as unifocal and unilateral stenosis or the irregularity of the large intracranial arteries of the anterior cerebral circulation [25]. Wintermark et al. subdivided FCA into three subtypes: FCA-inflammation type, including post-varicella arteriopathy, FCA-dissection type, which is typically associated with trauma, and FCA-undetermined type [25]. Arterial inflammation, whether infectious or postinfectious, may lead to arterial narrowing and thrombus formation on inflamed and damaged endothelium [25].

A multicenter VIPS study (Vascular effects of Infection in Pediatric Stroke) confirmed a robust association between clinical infection and childhood AIS: infection in the prior week conferred a 6.5-fold risk of AIS, with almost half of cases consistent with recent primary herpes virus infection (HSV 1) [26]. In a Canadian survey, the risk of stroke after varicella in childhood was estimated to be 1:15,000 [27]. Varicella is linked to AIS via two specific conditions: post-varicella arteriopathy (FCA occurring within a year of chickenpox) and varicella vasculopathy, which is related to recent infection and confirmed by demonstrating intrathecal VZV antibody production or VZV DNA in the CSF [28,29,30]. Stroke is more often connected to varicella reactivation or a secondary replication [26,29]. Based on animal studies, it is believed that varicella viruses in post-varicella vasculopathy would travel retrograde from the face to the trigeminal ganglion during varicella, but instead of all viruses entering a latent state, some viruses would undergo another round of replication in the trigeminal ganglion and then be carried anterograde to the cerebral arteries [31]. Even though VZV antibodies were proven negative on CSF essays, we believe that varicella vasculopathy could have caused CRAO in our patient.

Despite extensive work-up, we did not find an underlying systemic condition for CRAO in our patient.

### 3.4. Antiphospholipid Antibodies in COVID-19 Infection

The association of COVID-19 and aPL is established but somewhat unclear. Frequent single LAC positivity (during the acute phase) is observed in COVID-19 infection. Triple aPL positivity and high aCL/aβ2GPI titers are rare [32]. Several cases of IgM aCL appearing with thrombosis during acute COVID-19 infection have been reported [32,33]. CRAO has been documented two to six weeks after the initial onset of COVID-19 symptoms [34,35,36]. However, most patients had additional underlying conditions like hypertension, obesity, coronary artery disease, and a history of hospitalization or intensive care unit stay [33]. A case regarding a child suffering from CRAO during an acute COVID-19 infection was reported by Abbati et al. [11]. In the described case report, the ocular symptoms emerged after a one-day fever with a positive nasal swab and PCR test for the SARS-CoV-2 antigen. Abbati et al. did not report aPL testing [11]. Even though we cannot exclude that, in our case, the patient would have been positive if a PCR test was conducted during the illness of the family member two months prior to the symptoms emerging, we consider the connection of COVID-19 and CRAO in this case unlikely.

## 4. Conclusions

This is the first case report of CRAO in a child with transient aPL elevation and acute chickenpox infection.

## Figures and Tables

**Table 1:** Main laboratory findings at admission.

Parameter	Value	Normal Range
Erythrocytes	3.66 × 10^12^/L	4.00–5.00 × 10^12^/L
Hemoglobin	102 g/L	109–138 g/L
Hematocrit	0.296 L/L	0.320–0.404
Leukocytes	5.5 × 10^9^/L	5.0–13.0
Thrombocytes	189 × 10^9^/L	150–450
Lymphocytes	58%	15–55%
CRP	3.5 mg/L	<2.8 g/L
INR	0.97	-
Fibrinogen	3.5 g/L	1.6–4.0
D-dimers	1363 µg/L	0–500 µg/L

CRP—C-reactive protein; INR—international normalized ratio.

**Table 2:** Additional laboratory findings.

Parameter	Value	Normal Range
Factor VIII	2.10	0.58–1.32
LAC	-	-
Free protein S	0.67	0.62–1.30
Protein C	0.84	0.64–1.25
IgM aCL	238.50 CU	<20
IgG aCL	9.60 CU	<20
IgG aβ2GP1	76.1 CU	<20

LAC—lupus anticoagulant; IgM aCL—IgM anticardiolipin antibodies; IgG aCL—IgG anticardiolipin antibodies; IgG aβ2GP1—IgG anti-β2-glycoprotein 1 antibodies.

## Data Availability

The original contributions presented in this study are included in the article. Further inquiries can be directed to the corresponding author.
